# Anthracycline-induced cytotoxicity in the GL261 glioma model system

**DOI:** 10.1007/s11033-020-06109-8

**Published:** 2021-01-02

**Authors:** Amber M. Tavener, Megan C. Phelps, Richard L. Daniels

**Affiliations:** Department of Biology, The College of Idaho, Caldwell, ID 83605 USA

**Keywords:** Glioblastoma, GL261, Doxorubicin, MTT, Comet assay, Flow cytometry

## Abstract

Glioblastoma (GBM) is a lethal astrocyte-derived tumor that is currently treated with a multi-modal approach of surgical resection, radiotherapy, and temozolomide-based chemotherapy. Alternatives to current therapies are urgently needed as its prognosis remains poor. Anthracyclines are a class of compounds that show great potential as GBM chemotherapeutic agents and are widely used to treat solid tumors outside the central nervous system. Here we investigate the cytotoxic effects of doxorubicin and other anthracyclines on GL261 glioma tumor cells in anticipation of novel anthracycline-based CNS therapies. Three methods were used to quantify dose-dependent effects of anthracyclines on adherent GL261 tumor cells, a murine cell-based model of GBM. MTT assays quantified anthracycline effects on cell viability, comet assays examined doxorubicin genotoxicity, and flow cytometry with Annexin V/PI staining characterized doxorubicin-induced apoptosis and necrosis. Dose-dependent reductions in GL261 cell viability were found in cells treated with doxorubicin (EC_50_ = 4.9 μM), epirubicin (EC_50_ = 5.9 μM), and idarubicin (EC_50_ = 4.4 μM). Comet assays showed DNA damage following doxorubicin treatments, peaking at concentrations of 1.0 μM and declining after 25 μM. Lastly, flow cytometric analysis of doxorubicin-treated cells showed dose-dependent induction of apoptosis (EC_50_ = 5.2 μM). Together, these results characterized the cytotoxic effects of anthracyclines on GL261 glioma cells. We found dose-dependent apoptotic induction; however at high concentrations we find that cell death is likely necrotic. Our results support the continued exploration of anthracyclines as compounds with significant potential for improved GBM treatments.

## Introduction

Glioblastoma (GBM) is an aggressive primary brain tumor characterized by rapid cell division, a high degree of invasiveness into healthy tissue, and quick resistance to chemotherapeutic agents. Its prognosis is poor, with median survival rates of less than 1 year and 5-year survival rates of less than 5% [1–3]. Barriers to treatment include tumor location (because of the extraordinary sensitivity of surrounding tissue) and the blood-brain barrier (which excludes many anti-tumor compounds) [4, 5]. GBM is treated using surgical resection, radiotherapy, and chemotherapy with temozolomide [1]. Though this multi-modal therapy has modestly improved patient outcomes in recent years, improvements to current approaches and/or alternative therapies are urgently needed[6].

Anthracyclines such as doxorubicin, epirubicin, and idarubicin represent possible alternatives to temozolomide-based chemotherapy. These cytotoxic compounds, derived from Streptomyces bacteria, are effective against a variety of cancers because of their ability to induce apoptosis in tumor cells [7, 8]. Anthracyclines are widely used, and are prescribed to more than 30% of breast cancer patients and more than 50% of all childhood cancer patients [9]. In particular, doxorubucin’s efficacy against solid tumors is well established, and it is listed as a World Health Organization (WHO) essential medicine [10]. However, anthracycline-based chemotherapeutic regimens are not currently used to treat intracranial solid tumors such as GBM, as anthracycline antibiotics are excluded by the blood-brain barrier [11]. Recently a number of strategies have been proposed to treat intracranial solid tumors with doxorubicin, including using drug delivery vehicles such as polyanhydride polymers or nanoparticles, liposomal-loading, physical disruption of the blood-brain barrier, or pharmacological manipulation of blood-brain barrier efflux pumps [6, 12–15].

In anticipation of improved delivery methods for doxorubicin and its analogs to the site of tumor growth, we investigated the cellular toxicity of selected anthracycline compounds on the GL261 cell line, a murine-derived model of high grade gliomas and astrocytomas such as GMB. This model has many similarities with the human disease phenotype, including irregularly shaped borders, many different types of cells with atypical nuclei, similar mitotic activity, and multiple areas of necrosis [16]. However, despite these strikingly similar features to human GBM, few studies have explored doxorubicin toxicity using GL261 cells. We hypothesize that anthracycline compounds will be toxic to GL261 cells in a dose-dependent manner. Furthermore, we explore whether cell death occurs via apoptosis or necrosis. An improved understanding of doxorubicin’s cytotoxic effects on glioma tumors will further advance the exploration of anthracycline-based GMB therapies.

## Materials and methods

### Cell culture

GL261 cells were obtained from the National Cancer Institute’s DCTD Tumor Repository (Frederick, MD). Adherent cultures were maintained in DMEM (w/ L-glutamine, 4.5 g/L glucose and sodium pyruvate; Corning) with 10% Fetal Bovine Serum (Atlanta Biologicals) and 1.0% antibiotics (Penicillin/Streptomycin; Sigma-Aldrich). Cells were incubated at 37.0 °C with 5.0% CO_2_ in 75 cm culture flasks. Cells were passaged by trypsinization and media was changed every 2–4 days at approximately 80% confluency.

### MTT assay

Briefly, MTT assays were performed using the Vybrant MTT Cell Proliferation Assay kit (Invitrogen). In our assays, 200 μL of cells at 5 × 10^4^ – 1 × 10^5^ cells/mL were plated in triplicate in a 96-well plate and incubated for 24 h. After 24 h, cell solution was removed and replaced with 200 μL of media (vehicle control) or test compounds, and incubated for 24 h. Following incubation, media was removed and replaced with MTT reagent. After the addition of MTT, cells were incubated for another 4 h, and the solution was removed and replaced with 100 μl DMSO to solubilize the formazan. After 10 min, plates were analyzed using a BioRad Benchmark Plus microplate reader at 570 nm.

### Comet assay

The manufacturer’s instructions were followed (Trevigen). Briefly, cells were treated with concentrations of 0, 0.2, 1, 25, or 125 μM doxorubicin dissolved in H_2_O in 6-well culture plates for 15–17 h. Cells were suspended in low melting point agarose and pipetted onto Comet Assay microscope slides. The slides were placed into an electrophoresis chamber set at 20 V for 35 min at 300–350 amps. Cells were then stained with SybrGold (a DNA stain; Invitrogen) and allowed to dry completely before viewing with fluorescent microscopy and capturing images at 400X using a Nikon Eclipse Ti-S epifluorescent inverted microscope and associated Nikon Digital Sight DS-U3 camera. Images and videos were analyzed using the included Nikon Elements software. DNA staining was quantified using a DNA damage scale of 0–3, with 0 being no signs of DNA damage (no DNA “tails”) and 3 being complete DNA damage (long DNA “tails”) according to the manufacturer’s recommendations.

### Flow cytometry

Apoptosis and necrosis were quantified by flow cytometry using a BD Biosciences FACSCalibur and 10,000 gated events were collected for each trial. Cells were treated with doxorubicin at concentrations of 0, 0.2, 1, 5, 25, or 125 μM for 14–20 h. Treated cells were centrifuged, washed in PBS, centrifuged again, washed in annexin V binding buffer, and then incubated with annexin V staining solution at room temperature in the dark according to the manufacturer’s instructions (Annexin V, Alexa Fluor 488 conjugate; Invitrogen). Directly prior to quantification, propidium iodide (PI) stain (Invitrogen) was added to the cells.

### Data analysis

Image analyses were performed using ImageJ (NIH) and Nikon Elements software (Nikon). Means were calculated by averaging raw absorbance data and then normalizing to the vehicle control mean value. Sigma Plot 13 (Systat Software, Inc.) and Microsoft Excel were used for data analysis and statistical testing. Half-maximal effective concentrations of doxorubicin (EC_50_) were calculated using a web-based calculator (https://www.aatbio.com/tools/ec50-calculator).

## Results

### Anthracyclines decrease GL261 cell viability in a dose-dependent manner

**Fig. 1:**
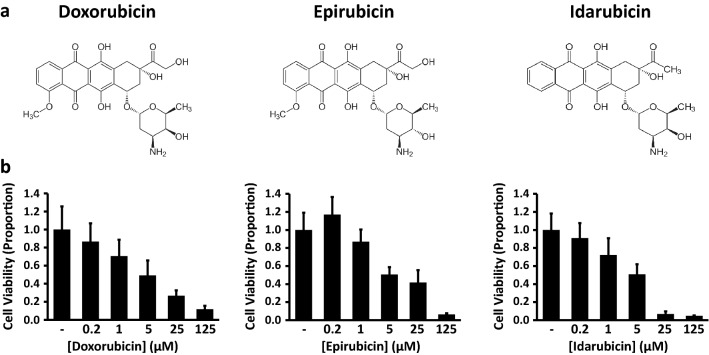
Anthracyclines decrease GL261 cell viability in a dose-dependent manner. (**a**) Chemical structures for doxorubicin, epirubicin, and idarubicin (**b**) Cell viability following 24-h treatments with vehicle control or test compounds at the indicated concentrations (MTT assay). Values indicate mean ± standard error (*n* = 7 for doxorubicin, epirubicin; *n* = 6 for idarubicin)

Anthracyclines are tetracyclic compounds used to treat a variety of cancers, and include the compounds doxorubicin, epirubicin, and idarubicin (Fig. 1a; images are public domain from Wikimedia Commons). We examined the effects of these anthracyclines on GL261 cells using the MTT cell viability assay, a colorimetric method where 3-(4,5-dimethylthiazol-2-yl)-2,5-diphenyltetrazolium bromide (MTT) is reduced to formazan by metabolic enyzmes. The resulting product causes the media surrounding the cells to change from yellow to purple, thereby providing an indirect measurement of cell viability. We hypothesized that each anthracycline would decrease cell viability in a dose-dependent manner. Cells were treated for 24 h with either vehicle control or the test compound at concentrations of 0.2, 1.0, 5.0, 25, or 125 μM (Fig. 1b). Our results supported our hypothesis, with increasing drug concentrations leading to corresponding decreases in cell viability. For doxorubicin, the above treatments reduced cell viability from a normalized vehicle control value of 1.0 ± 0.26 to 0.87 ± 0.20, 0.71 ± 0.18, 0.49 ± 0.17, 0.27 ± 0.06, and 0.12 ± 0.04, respectively (*n* = 7, results given in mean ± standard error). Similarly, epirubicin altered cell viability from a normalized vehicle control 1.0 ± 0.19 to 1.17 ± 0.19, 0.87 ± 0.13, 0.51 ± 0.08, 0.42 ± 0.14, and 0.06 ± 0.01 (n = 7). Cells exposed to idarubicin had decreased viability as well, from a normalized vehicle control 1.0 ± 0.18 to 0.91 ± 0.17, 0.72 ± 0.19, 0.51 ± 0.11, 0.07 ± 0.03, and 0.05 ± 0.00 (*n* = 6). A Kruskal-Wallis one-way Analysis of Variance (ANOVA) revealed a significant difference among the various doxorubicin treatments (*p* = 0.004), and likewise among the epirubicin (*p* < 0.001) and idarubicin (p < 0.001) treatments. Half-maximal effective concentrations (EC_50_) were calculated as follows: doxorubicin (4.9 μM), epirubicin (5.9 μM), idarubicin (4.4 μM). Thus we found that each anthracycline decreased cell viability in a dose-dependent manner.

### Doxorubicin treatment leads to a dose-dependent increase in DNA damage

**Fig. 2:**
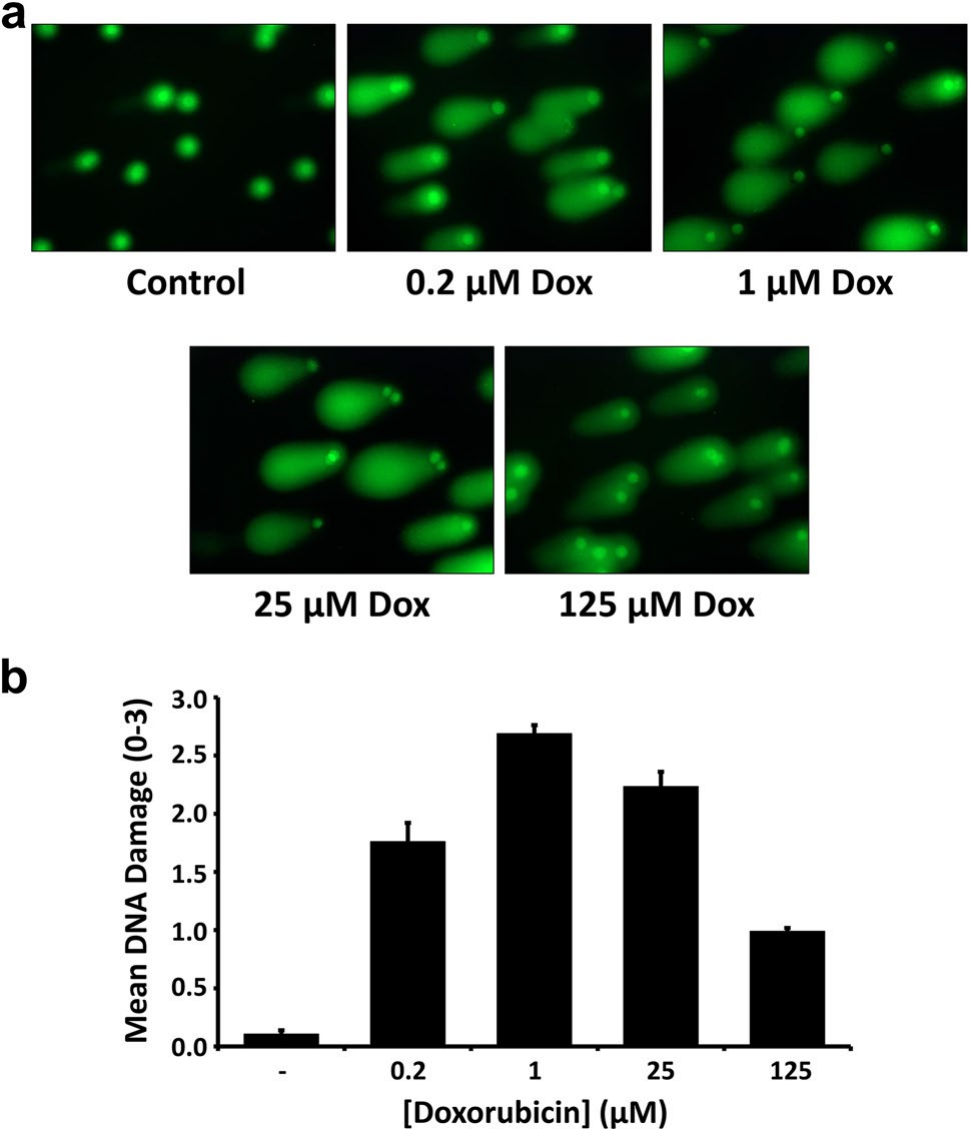
Doxorubicin treatment leads to a dose-dependent increase in DNA damage. (**a**) Photomicrographs (400X) depict SYBR® Gold fluorescent staining of GL261 cells following electrophoresis through low melting point agarose (Comet Assay). Prior to imaging, cells were treated for 15–17 h with vehicle control or doxorubicin at the indicated concentrations (**b**) Quantification of comet assay results. DNA Damage scores were calculated based on comet tail length, and results are reported as mean ± standard error (*n* = 5)

To learn more about the nature of the decrease in cell viability, comet assays were used to assess the degree of DNA damage in GL261 cells following the application of doxorubicin (Fig. 2). DNA damage is a hallmark of apoptosis, and thus we hypothesized that doxorubicin will damage DNA and allow it to be detected extracellularly [17, 18]. Electrophoresed GL261 cells were stained with SybrGold fluorescent DNA stain, and scored based on fluorescence. DNA staining was quantified using a DNA damage scale of 0–3, with 0 being no signs of DNA damage (no or very short “comet tail”) and 3 being complete DNA damage (long “comet tail”) according to the kit manufacturer’s recommendations. We found that doxorubicin is associated with dose-dependent apoptosis in GL261 cells as indicated by DNA fragmentation. At concentrations of 0, 0.2, 1, 25, and 125 μM doxorubicin, mean DNA damage scores were recorded of 0.11 ± 0.03, 1.76 ± 0.16, 2.69 ± 0.07, 2.23 ± 0.13, and 0.99 ± 0.03, respectively (results given as mean ± standard error, *n* = 5). A One-way Analysis of Variance (ANOVA) revealed a significant difference among all treatment groups (*p* < 0.001), and post-hoc testing (Holm-Sidak) showed significant differences in all pairwise comparisons (each with *p* < 0.01). Therefore, we find that doxorubicin is associated with dose-dependent DNA damage in GL261 cells.

### Doxorubicin treatment leads to apoptosis and necrosis in GL261 cells

**Fig. 3:**
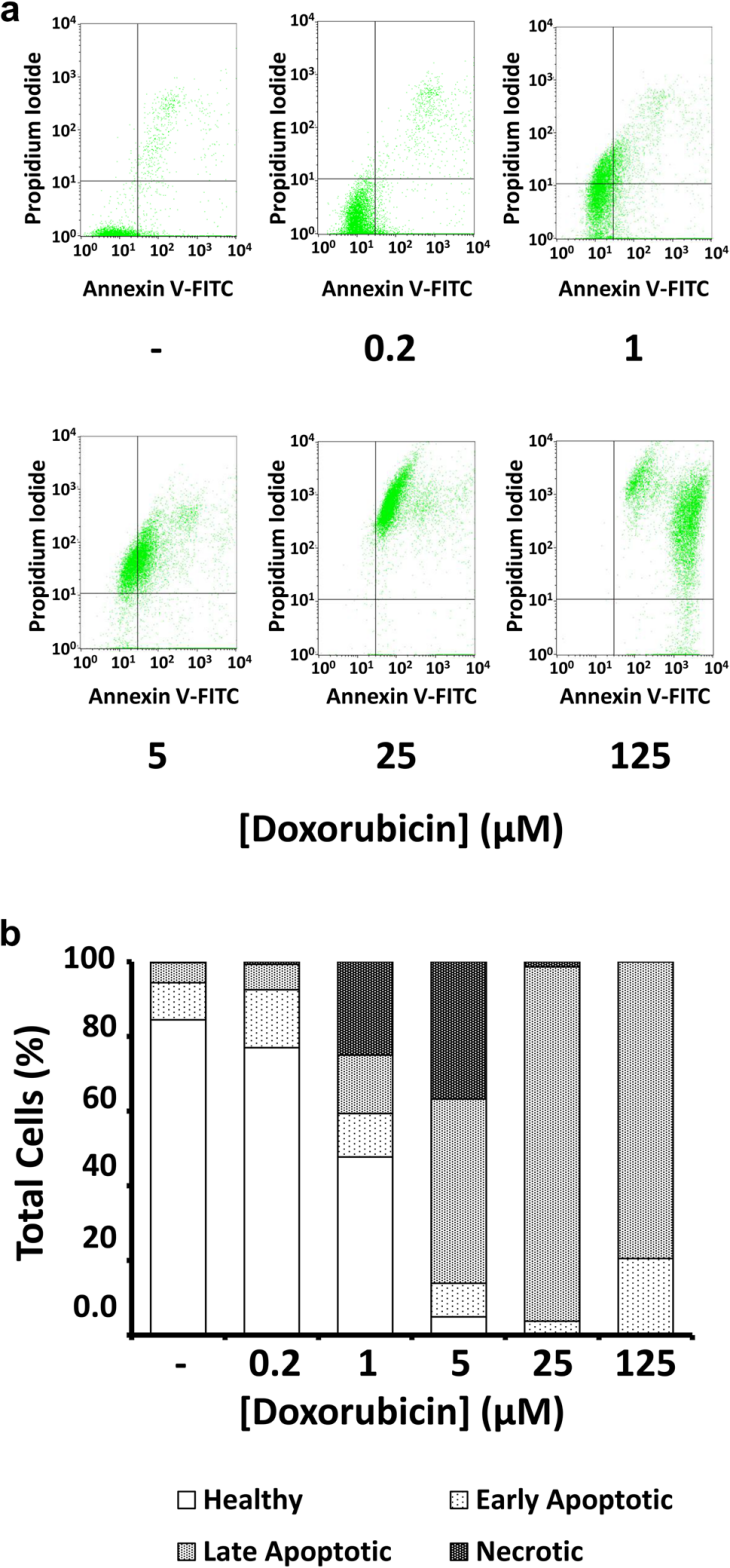
Doxorubicin treatment leads to apoptosis and necrosis in GL261 cells. (**a**) Flow cytometry analysis of GL261 cells treated for 15–17 h with doxorubicin, stained with Annexin V-FITC, and counterstained with propidium iodide (PI). Dot plots show positioning of quadrants distinguishing healthy cells (lower left; Annexin V- / PI -), early apoptotic cells (lower right; Annexin V+ / PI -), late apoptotic cells (upper right; Annexin V+ / PI+), and necrotic cells (upper left; Annexin V- / PI+). (**b**) Bar graph showing proportions of healthy, apoptotic, and necrotic cells after the indicated doxorubicin treatments

Lastly, we used flow cytometry to investigate whether doxorubicin-mediated cytotoxic effects on GL261 cells were primarily due to apoptosis or necrosis (Fig. 3). Cells were stained with Annexin V-FITC and propidium iodide. Annexin V is a protein that binds to phosphatidylserine, a membrane phospholipid normally found in the inner leaflet of the cell membrane but found in the extracellular face in apoptotic cells; propidium iodide is a DNA-specific stain that is membrane impermeant, and will thus only stain dead cells or during late apoptosis after the membrane has been severely compromised [19]. Thus cells can be categorized as healthy (Annexin V- / PI -), early apoptotic (Annexin V+ / PI -), late apoptotic (Annexin V+ / PI+), or necrotic (Annexin V- / PI+) based on their fluorescence profiles. Following treatment with 0, 0.2, 1.0, 5.0, 25, and 125 μM doxorubicin, we analyzed 10,000 cells per treatment and found the percentage of healthy cells decreased from 84.4% to 77.0%, 47.7%, 4.9%, 0.4%, and 0.1% over this range. We found few early apoptotic cells in untreated controls (10.0%), but as doxorubicin concentrations increased the percentage of early apoptotic cells rose to 15.6%, 11.6%, 9.0%, 3.3%, and 20.5%, respectively. Likewise, the percentage of late apoptotic cells also increased with increasing doses of doxorubicin, from 5.4% to 6.8%, 15.7%, 49.3%, 95.0%, before dropping to 79.5% at the highest dose. We found the percentage of necrotic cells at these same concentrations to be 0.3%, 0.7%, 25.0%, 36.8%, 1.4%, and 0.0%, respectively. These data are summarized in Fig. 3b. We calculated the half-maximal effective concentration (EC_50_) from these dose-response relationships in several ways. First, we found doxorubicin to have an EC_50_ of 5.2 μM regarding its ability to induce apoptosis (early and late were considered together). However, doxorubicin was found to be more potent (EC_50_ = 1.2 μM) if calculated considering solely its cytotoxic effect on cells (that is, its ability to cause apoptosis or necrosis). Thus we found that doxorubicin had a dose-dependent apoptotic and necrotic effect on GL261 cells.

## Discussion

Doxorubicin is one of the most important drugs available to treat solid tumors and childhood cancers [9]. Doxorubicin intercalates into DNA and subsequently interferes with enzymatic binding, including disrupting topoisomerase activity that is responsible for DNA replication [7, 20]. Here we found that anthracyclines were cytotoxic to GL261 cells in a dose-dependent manner. Our methods led to insights into whether doxorubicin leads to cell death via apoptosis or necrosis. Interestingly, comet assays showed that doxorubicin-induced DNA damage was concentration-dependent, with observed damage decreasing at the highest doses. One explanation for this is that apoptosis is initiated at low doses, while at higher concentrations cell death is necrotic. We would expect to find support for this in our flow cytometry data, where we see a rise in both apoptosis and necrosis following doxorubicin treatments up to 5 μM. However, we find little necrotic cell staining at higher doses. It is possible that at the highest doses many cells may had already died, as cell counts were far lower in experimental treatments with higher doxorubicin concentrations. Thus we may not be measuring cell death via necrosis at high doses in our flow cytometry data because these cells are no longer present (in other words, at high doses most cells have already died, and the ones that are left are nearly all apoptotic). Another source of uncertainty in these data is the similar fluorescence emission spectra of propodium iodide (617 nm) and doxorubicin (595 nm). It is possible that some cells with high doxorubicin autofluorescence are mistaken for late apoptotic rather than early apoptotic. In any case, the half-maximal effective dose for doxorubicin was measured at 1.2 μM in terms of cytotoxicity, but 5.2 μM in terms of apoptosis. Together, our data show that lower concentrations of doxorubicin are associated with apoptotic cell death, while higher concentrations are associated with reduced cell numbers and necrotic cell death. More work is necessary to establish the extent to which cell death is apoptotic or necrotic at a given dose and exposure time.

Our results are broadly consistent with previous investigations on doxorubicin cytotoxicity. In Hela cells, apoptosis and genotoxicity is initiated within less than 2 h following 9 μM doxorubicin treatment (as measured by caspase 3 gene expression and γH2AX phosphorylation) [7]. Doxorubicin-induced cytotoxicity has been characterized in a number of cell types, with half-maximal effects following 24–48 h treatments ranging from 0.5–10 μM when examined in M059j and M059k human glioblastoma cells, hct116 colon cancer cells, MCF-10F, MCF-7 and MDA-MB-231 breast cancer cells [8, 21, 22]. In our review of the literature, we found a single study which reported the effects of doxorubicin over a longer time-frame on the GL261 cell line as an ancillary finding, with half-maximal effects on GL261 cell viability after 72 h of 0.2 μM doxorubicin treatment [23]. It should be noted that the drug responses of cells grown in culture can vary with culture conditions[24]. Thus, our results may in part reflect the adherent phenotype of our GL261 cell culture model. Still, the findings reported here are consistent with previous results in a number of cell types.

A recent study used sonication-based disruption of the blood-brain barrier to deliver doxorubicin to experimentally-placed GL261 tumors in mice, resulting in slowed disease progression and increase of survival [25]. It is interesting to note that CNS doxorubicin concentrations in that same study were measured at 0.2 and 0.3 μM, suggesting that newly-developed methods are capable of delivering anthracyclines to CNS tumors at concentrations that we found to be cytotoxic.

Taken together, our results show that anthracyclines represent a promising alternative to current temozolomide-based therapies for GBM and that further research is warranted that explore their use as CNS anti-tumor compounds.

## Data Availability

All data will be made available upon request.
